# Detectability of cytokine and chemokine using ELISA, following sample-inactivation using Triton X-100 or heat

**DOI:** 10.1038/s41598-024-74739-0

**Published:** 2024-11-05

**Authors:** Erica Hofer Labossiere, Sandra Gonzalez-Diaz, Stephanie Enns, Paul Lopez, Xuefen Yang, Biniam Kidane, Gloria Vazquez-Grande, Abu Bakar Siddik, Sam Kam-Pun Kung, Paul Sandstrom, Amir Ravandi, T. Blake Ball, Ruey-Chyi Su

**Affiliations:** 1 JC WILT Infectious Disease Research Center, National Microbiology Laboratories, Public Health Agency of Canada, Winnipeg, MB Canada; 2 Department of Medical Microbiology & Infectious Diseases, University of Manitoba, Winnipeg, MB Canada; 3 Section of Thoracic Surgery, Department of Surgery, Health Sciences Centre, University of Manitoba, Winnipeg, MB Canada; 4 Department of Physiology and Pathophysiology, Rady Faculty of Health Sciences, Max Rady College of Medicine, University of Manitoba, Winnipeg, MB Canada; 5 Department of Biomedical Engineering, Price Faculty of Engineering, University of Manitoba, Winnipeg, MB Canada; 6 Section of Critical Care, Department of Medicine, University of Manitoba, Winnipeg, MB Canada; 7 Department of Immunology, University of Manitoba, Winnipeg, MB Canada; 8 Cardiovascular Lipidomics, Institute of Cardiovascular Sciences, St. Boniface Hospital, Max Rady College of Medicine, University of Manitoba, Winnipeg, MB Canada

**Keywords:** Triton X-100, Cytokine/chemokine, Heat-inactivation, Lung aspirate, Nasopharyngeal swab, ELISA, Immunology, Microbiology, Molecular biology, Biomarkers, Diseases, Molecular medicine

## Abstract

Clinical samples are routinely inactivated before molecular assays to prevent pathogen transmission. Antibody-based assays are sensitive to changes in analyte conformation, but the impact of inactivation on the analyte detectability has been overlooked. This study assessed the effects of commonly used inactivation-methods, Triton X-100 (0.5%) and heat (60 °C, 1 h), on cytokine/chemokine detection in plasma, lung aspirates, and nasopharyngeal samples. Heat significantly reduced analyte detectability in plasma (IL-12p40, IL-15, IL-16, VEGF, IL-7, TNF-β) by 33–99% (p ≤ 0.02), while Triton X-100 minimally affected analytes in plasma and nasopharyngeal samples (11–37%, p ≤ 0.04) and had no significant impact on lung aspirates. Structural analysis revealed that cytokines affected by heat had more hydrophobic residues and higher instability-indices. As the protein-detectability was affected differently in different sample types, the sample environment could also influence protein stability. This underscores the importance of selecting the most suitable inactivation methods for clinical samples to ensure accurate cytokine/chemokine analysis in both clinical and research settings.

## Introduction

Host cells respond to viral infection with secretion of cytokine and chemokine, and subsequent recruitment and activation of immune cells. Cytokines modulate cellular activity and function, while chemokine facilitates the recruitment of immune cells, such as monocytes/macrophages, neutrophils, T-lymphocytes, Mast cells, and Eosinophil^1,2^. These responses to viral infection are often sufficient to resolve viral infection without medical treatment. Ninety-three percent of patients who had an infection due to respiratory virus SARS-CoV-2 recovered without requiring hospitalization developed protective immunity against future infection^3^. However, in severe cases, the host response can be insufficient or inappropriate in eliminating the virus^4^. Of the hospitalized patients, 20% experienced severe symptoms requiring intensive care admission^5^. It is critical to determine the cytokine/chemokine response that lead to a protective immune response, as well as the cytokine/chemokine profile that leads to failure in controlling viral infection or tissue damage.

To examine these critical immune mediators, the clinical samples from the infected patients need first being inactivated to prevent the spread of infection to sample-handlers and to increase access to instruments located outside of the CL3 lab. The most commonly collected clinical samples is blood, due to the ease of access. In this study, blood and samples from different parts of respiratory tract, the main site of transmission were collected from SARS-CoV-2 positive patients. This consisted of endotracheal tube aspirate (ETTA), transport media for nasopharyngeal (NP) swab, and plasma. Methods have been tested for proper inactivation of virus. While the efficacy of various methods for inactivating the viral infectivity has been documented^6^, little work has been performed to determine the effects of these methods on the specificity and sensitivity of the molecular assays in the detection of these immune mediators. *Patterson *et al*.* demonstrated that 0.5% Triton X-100 (t-Octylphenoxy-polyethoxy-ethanol) and heat could efficiently inactivate SARS-CoV-2 in clinical samples^6–8^. This study further examined and quantified the impacts of 0.5% Triton X-100 and heat, commonly used inactivation method, on the detection of cytokine/chemokine in blood, the most collected sample type, in addition to other clinical sample types.

Antibody-based molecular assays are highly sensitive to changes in protein conformation^9,10^. Should the inactivation method change the conformation of the analytes of interest, the sensitivity of detection could be affected and often, decreased or lost^9–11^. Heat inactivation is an accessible, eco-friendly, and cost-effective method of inactivating infectious agents and is often used in study of lipids, metabolites, and nucleic acids^12,13^. As temperature increases in the samples, kinetic energy in molecules can disrupt hydrogen bonding and the dispersion forces in proteins. Ultimately, inactivating the virus present by denaturing the structure of its constituent proteins, which interferes with both viral attachment and replication^7,8^. Coronaviruses have shown a reduction of viral infectivity ≥ 4.0 log_10_ when incubated at 60 °C for 30 min^8^. However, heat’s effects on proteins are not specific to viral proteins, and can affect cytokines and other proteins in the sample. Triton X-100 is a nonionic surfactant that has been widely used to permeabilize cellular and viral membrane for downstream clinical and research applications^14^. In SARS-CoV-2 research, 5, 2, 1 and 0.5% Triton X-100 has been shown to be effective in inactivating viral replication. This study has chosen 0.5% concentration because 0.5% Triton X-100 has been shown to have minimum impact on protein metabolites^15,16^ and has been routinely used to inactivate HIV-1 culture for downstream cytokine/ chemokine analysis. Heat, among many other physical, chemical and energetic methods and combinations thereof is also commonly used to inactivate virus^17^. Inactivation of SARS-CoV-2 could be achieved with different conditions of heat-treatment: 56 °C for 30 min, 60 °C for 60 min, or 65 °C for 15 min^8,13,17–20^. Heat denatures viral capsid and envelope proteins leading to alteration of specific structures/epitopes necessary for the virus to recognize and bind to host cellular proteins, thereby inhibiting viral replication. Exposure to 60 °C heat for 60 min has been shown to denature Ebola, Marburg viruses, Alkhurma hemorrhagic fever virus, as well as Chikungunya and Ross River virus^21^, and has been the standard viral inactivation protocol in genomic sequencing service core (National Microbiology Laboratories) and metabolomics core (The Metabolomics Innovation Centre). Hence, this study chose to examine the impacts of the heat-inactivation treatment, 60 °C for 60 min.

Although heat has been commonly used to inactivate clinical samples, only few studies examined the effect of heat -treatment on the detection of cytokine/chemokine and most of these studies focused on the inactivation of plasma or serum samples^20,22^. Exposing plasma samples to 56 °C heat for 60 min reduced the detection of protein analytes by 307 + /- 563 fold (IL-6R p-value = 6. 7E-08) and 88.2 + /− 10.2 fold (MMP-10, p-value = 2.03E-07)^22^. In a separate study, exposure of blood samples to 56 °C heat for 30 min significantly reduced the detection of TSH, T3, FT4, FT3, AFP, NSE, CYFRA21-1, IL-6, IL-10, IL-2R and TNF-α^20^. Yet, the effect of heat on lung fluids or nasopharyngeal samples, which are key to understand respiratory infections and treatment management still require further investigation.

This study found that both 0.5% Triton X-100 and heat-inactivation altered the sensitivity of detection of several cytokine/chemokine analyzed but with different magnitude of impacts. The 0.5% Triton X-100 affected the detection of analytes in almost only the nasopharyngeal swab, but to a moderate degree. In contrast, heat had much impact. Findings in this study are of critical values for research and clinical diagnosis or treatment-management. Clinically, when one of the effected cytokines is assessed as an important biomarker in AIDS, tumors, infections, or autoimmune disorders, this could result in incorrect diagnosis if the improper inactivation method is used^23–28^.

## Materials and methods

### Ethics, inclusion and exclusion criteria and consent

This study is approved by both the University of Manitoba Research Ethic Board [HS24126 (B2020:073)] and the Public Health Agency of Canada (REB 2020-036P). This is an ad hoc analysis of samples and data collected for the approved COVID-19 research.

We included patients admitted to the intensive care units (ICU) of Health Sciences Center in Winnipeg, MB, Canada during March-July 2021, enrolling ICU COVID-19 patients who were diagnosed with acute respiratory distress syndrome (ARDS) according to the Berlin Criteria and required intubation (diagnosis details included in greater detail in following publication)^29^.

We obtained deferred informed consent from the legally authorized representative of all included patients within 3 days of intubation, following the declaration of Helsinki protocols. The day 1 samples from the patients, whom we were unable to obtain informed consent by day 3, were discarded, and the patients were removed from the study.

### Sample collection and storage

Whole blood using EDTA-coated tubes (BD Biosciences, USA), endotracheal tube aspirates (ETTA), and Nasopharyngeal (NP) swabs (COPAN, Italia) were collected. The NP swabs were placed in Universal Transport Medium (UTM, COPAN, Italia) after collection.

Blood samples were centrifuged at 250xg for 8 min. Plasma sample (upper layer) was collected into a new tube and was spun again to remove cellular debris. Cells from the NP swabs immerged in the UTM were dislodged by mixing on a vortex for 30 s. The UTM was centrifuged at 250xg for 8 min to separate the cells from soluble factors. The supernatant containing soluble factors was collected. ETTA sample was mixed vigorously for 30 s to dissociated mucus on a vortex. After mixing, the sample was passed through a 350-micron then a 100-micron filter to remove mucus clumps. The sample was then centrifuged at 250xg for 8 min to separate cells and soluble factors. The supernatant was collected. Aliquots of all samples were stored at −80 °C until analysis.

### Sample inactivation

#### Thermal inactivation method

Samples were thawed on ice. Thawed sample tubes were placed in a 60 °C heating block and submerged past the sample level for 60 min. Following inactivation, the samples were gently mixed and centrifuged to collect any inner condensation and to remove potential aggregates or debris.

#### Triton X-100 inactivation method

After samples were thawed on ice. They were then centrifuged to bring all sample droplets to the bottom of the tube. Triton X-100 (Catalog #T8787, MilliporeSigma Canada Ltd, Canada) was added to each sample to reach a final concentration of 0.5%. Samples were mixed briefly and incubated for 15 min at room temperature before use.

### Meso scale discovery (MSD) multispot assay

Cytokines and chemokine were measured using Meso Scale Discovery Multi-Spot Assay V-plex Cytokine Panel 1 (Human) Kit (Catalog #K15050D), V-plex Chemokine Panel 1 (Human) Kit (Catalog #K15047D), V-plex Proinflammatory Panel 1 (Human) Kit (Catalog #K15049D) and V-plex Th17 Panel 1 Human) Kit (Catalog #K1505D) (Meso Scale Diagnostics LLC, Rockville, USA), following the manufactures’ detailed protocol, from each V-plex panel. Each assay was performed with two technical replicates for each sample (with CV < 15%), with a dilution factor (DF) of 2 for Cytokine and Pro-inflammatory Panels or a DF of 4 for Chemokine and the Th17 Panels. A calibrator was included in each experiment and the standard curve for each assay was generated by eight serial dilutions with a DF of 4 and in 2 technical duplicates. Sample-dilutions were pre-made and well mixed by shaking at 700 rpm on a vortexor for ≥ 5 min. Dilution factor is specific to each sample-type and unique to the MSD panel that was used. Diluted samples were mixed by a quick vortex before being added into assay wells. Plates were washed with 155 µL Wash Buffer (PBS, 0.05% Tween-20) per well. The assays were read on the Model 1300 MESO® QuickPlex SQ 120MM (Meso Scale Diagnostics LLC, Rockville, USA). Due to the wide physiological range of IL-8, its low and normal physiological ranges were detected using chemokine panel 1 kit, whereas its high concentration was detected using pro-inflammatory panel 1.

### Data analysis

Concentration values that were below the assay’s limit of detection (LOD) were assigned a value of half the lower LOD to include them in analyses. Mean concentrations that had a coefficient of variation higher than 20% were not included in statistical analysis. All statistical tests were performed by GraphPad Prism version 9.5.1 for Windows (GraphPad Software, San Diego, USA). Non-parametric Wilcoxon matched-pairs signed-rank t tests, using Pratt’s method, were performed based on the normality of the dataset. Statistical significant difference established as (*) p ≤ 0.05; (**) p ≤ 0.01; (***) p ≤ 0.001; (****) p < 0.0001.

The *delta concentration*($\Delta \overline{x}$) was calculated by subtracting the median concentration from untreated control samples (pg/mL) from the median of $\Delta$concentration of heat-treated or Triton X-100 treated samples (pg/mL), using the following equation (mean or median depending on normality test):

$$\Delta \overline{X} \, = \, \overline{X}_{treatment} - \overline{X}_{control}$$

Stability of cytokines were assessed by calculating *percentage of change* ($\Delta \%$) using mean/median concentration values, as per the following equation. p-values of percent change were calculated using the method of Pratt two-tailed Wilcoxon Signed Rank Test.

$$\Delta \% = 100\% *\left( {\overline{X}_{treatment} - \overline{X}_{control} } \right)/\overline{X}_{control}$$

### Cytokine structure analysis

Amino acid sequence of each cytokine was extracted from the UniProtKB database^30^ to investigate the protein features that might contribute to the instability of its structure. Several features of the protein could affect its stability in solution; hence, several features were chosen to be analyzed in this study. These included isoelectric point, net charge of the protein, instability index (II), grand **av**erage of hydropathicit**y** (GRAVY), estimated in vitro half-life (hour), amino acid composition scores, and secondary and tertiary protein structure. Online application, isoelectric point calculator (IPC) was used to generate isoelectric point and net charge of the protein at physiological pH^31^. The ranges of amino acid chain sequence position (the polypeptide chain in the mature protein as denoted by UniProt) were used to generate the instability index (II) GRAVY, estimated in vitro half-life (hour), and amino acid composition scores, using the online Expasy ProtParam program^32–34^. Secondary and tertiary protein structure visualization was performed using AlphaFold^35^ and Swiss-Model with recommended parameters^36–44^.

## Results

### Heat-inactivation changed detection levels of several cytokines/chemokine in all samples types

**Fig. 1:**
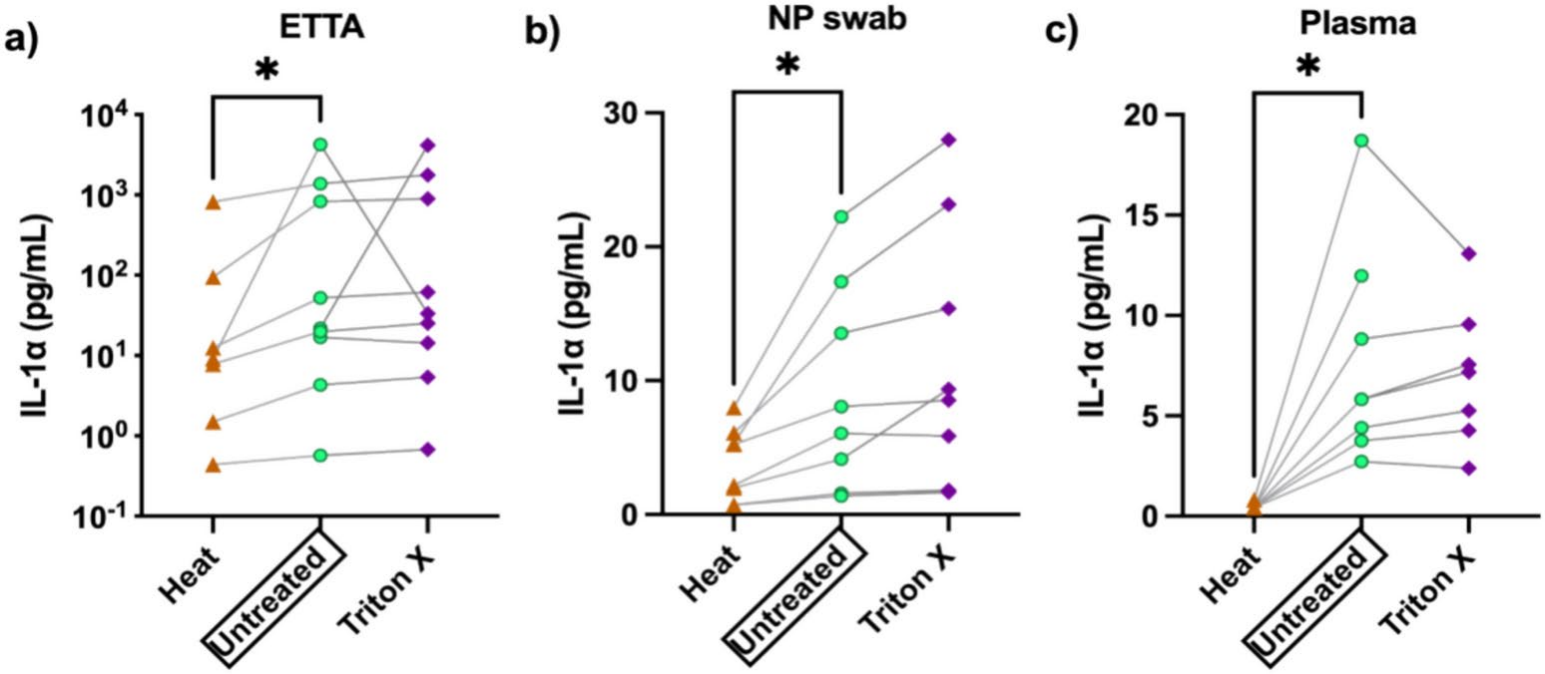
Detection of IL-1α in heat- and Triton X-100- treated versus untreated control samples from (**a**) ETTA, (**b**) NP Swab, and (**c**) Plasma. Paired aliquots of the same sample (connected with a line) were inactivated by exposure to 60 °C for 1 h (▲), or Triton X-100 (0.05%, ♦), or left untreated on ice (○). Some samples were unpaired due to unavailability of sample to be tested in all three conditions. One sample in 1c is missing the Triton X-100 treated aliquot. The levels of IL-1α detected in these samples were assayed and compared using paired t-test: ‘*’ denotes p < 0.05, ‘**’ denotes p < 0.005.

Exposure to heat, 60 °C for 60 min had undesirable impact on the detection of IL-1α in all 3 sample types, the plasma, lung fluid (ETTA) and NP swab (Fig. 1). In contrast, exposure to 0.5% Triton X-100 had no significant effect on the IL-1α detectability in all 3 sample types. For most samples and analytes studied, the heat-treatment induced decreases in detection remained within the biological variation of the untreated samples. For example, IL-1α levels detected in ETTA and NP-swab that were inactivated by heat (orange symbols) were in the lower range of the biological variation of the untreated samples (green symbols) (Fig. 1a,b). Significant reduction outside of the biological variation of the untreated samples was observed only in heat-inactivated plasma samples (Fig. 1c). The IL-1α data in Fig. 1 is representative of the affected analytes examined in this study, as listed in Table S1a. Not all analytes affected by heat were also affected by Triton X-100 and vice versa. However, analytes that were affected were not affected to the same extent; biological variation was observed in the ETTA and UTM-NP swab samples (Fig. 1a,b), but not plasma samples (Fig. 1c). Five of the total 36 analytes examined in the heat-treated plasma samples exhibited significantly reduced detected levels that are below the biological range of the untreated samples. It suggests that heat-inactivation has greater impact on the analytes in plasma samples, independent of the biological sample variation, evident of a tight coefficient variation (CV).

**Fig. 2:**
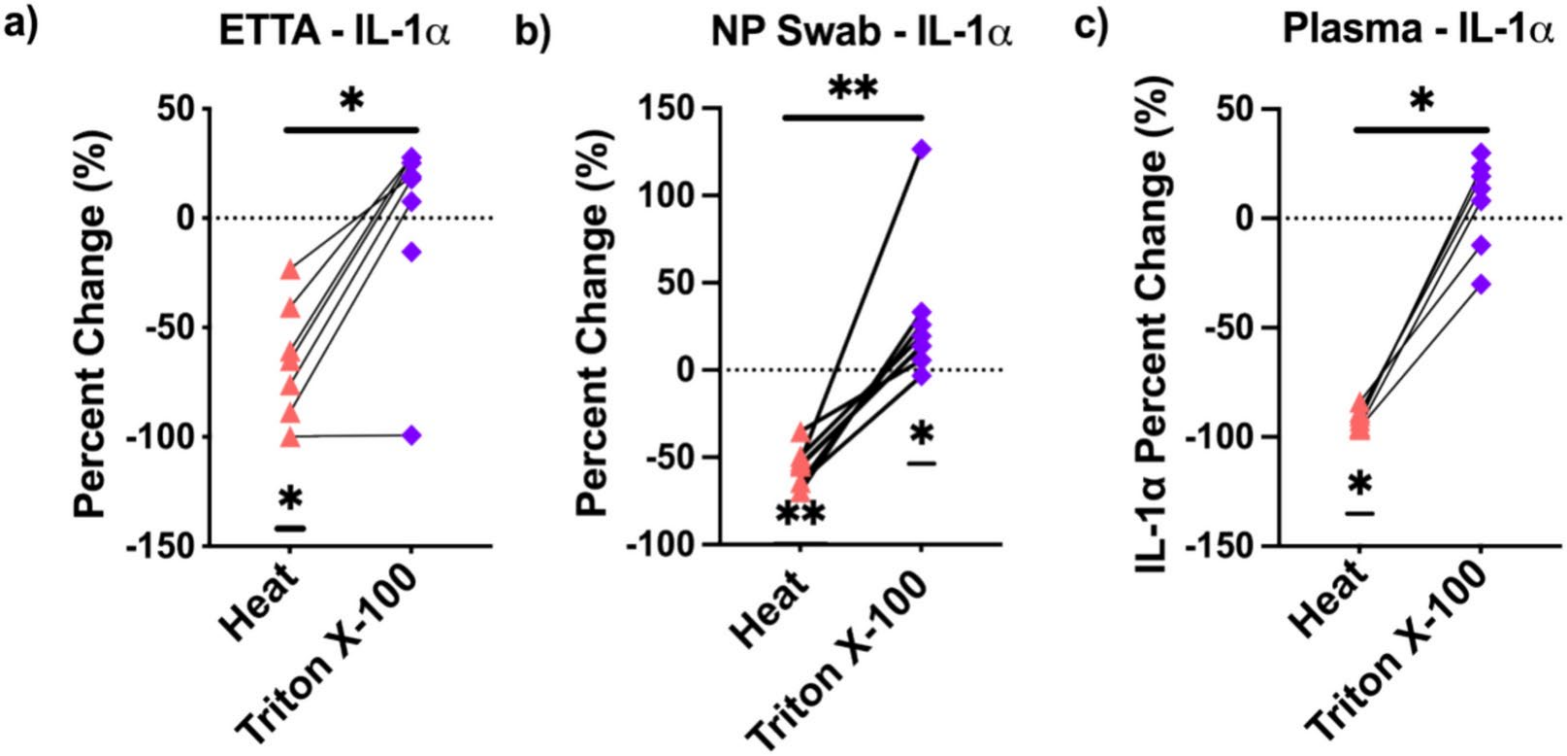
The magnitude of effects of heat and Triton X-100 on the detection of IL-1α measured in percentage changes from the untreated samples. Paired aliquots of the same sample (connected with a line) were inactivated by exposure to 60 °C for 1 h (▲), or Triton X-100 (0.05%, ♦), or left untreated on ice. Some samples were unpaired due to unavailability of sample to be tested in all three conditions. The magnitude of effects on IL-1α detection in percentage was tested against a hypothetical value ‘0’ (dashed line on the graph) using “Wilcoxon Signed Rank Test”. The dashed line represents ‘0%’ deviation from the detected level of cytokine/chemokine in the untreated samples and is calculated as [(S_treated_−S_untreated Ctrl_)/ S_untreated Ctrl_ × 100%].The p-values were shown near x-axis. The difference in the magnitude effects between the two inactivation-methods was tested using "Wilcoxon matched-pairs signed rank test". ‘*’p < 0.05, ‘**’p < 0.005.

To determine whether the inactivation methods had similar level of impact on the affect analytes within a sample type, the magnitude of the effects by the inactivation was calculated as percentage (%) decrease or increase from the untreated control sample (0%, dashed line). Figure 2 shows that heat, 60 °C for 60 min had varying magnitude of effects on IL-1α detection in ETTA sample (−23 to −100%), and smaller range of effects on IL-1α detection in NP swab (−35 to −69%) or plasma samples (−84 to −96%). While the magnitude of effects of heat-treatment on IL-1α detection was significantly different than 0% in all 3 sample-types, the effect of Triton X-100 on IL-1α detection was only significantly increased in the NP swab samples (*p = 0.016), with a median of + 17%, which is a small percentage when compared to the effects of heat-treatment (−55%, **p = 0.003). The contrasting effects of heat vs. Triton X-100 on IL-1α detection were similarly observed in some of other affected analytes (Table S1a).

**Fig. 3:**
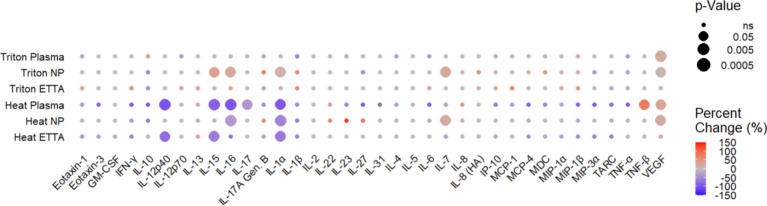
Summary of the effects of heat and Triton X-100 on the detectability of cytokine/chemokine, expressed as percent change, in ETTA, NP Swab, and plasma samples. Each dot represents a cytokine or chemokine in a clinical sample treated by either heat 60 °C for 60 min or 0.5% Triton X-100. Dot colour-scale is based on median % value. Using untreated sample as reference, decrease or increase in % of cytokine/chemokine detected in the treated samples were shown in blue or red color, respectively. Grey color depicts ‘no difference’ from the untreated reference sample. Exact percent change values can be found in Table S2. The size of the dot represents log_10_ p-values. The larger the dot in diameter, the lower the p-values (Pratt two-tailed Wilcoxon Signed Rank Test).

The magnitude (% changes) and significance (p-value) of the impact due to heat-treatment or Triton X-100 on the detection of the 36 analytes assessed in 3 different sample-types were summarized in a colored dot plot below (Fig. 3). Overall, the effect of 0.5% Triton X-100 (the top three rows) was mostly associated with increased detection of the analytes. Moreover, 0.5% Triton X-100 affected preferentially the analytes in the NP swab sample. Approximately 11–37% more of IL-15, IL-16, IL-1α, IL-7, and VEGF (5 of 36 cytokine/chemokine) were detected (*p < 0.05, Fig. 3). Besides its effects on VEGF in plasma, 0.5% Triton X-100 had no significant impact on the detection of the other 35 analytes in plasma or ETTA. In contrast, heat-inactivation (the bottom three rows) significantly reduced the detection of several analytes by great magnitudes (blue) in all 3 sample-types. More analytes in the plasma sample were affected. Both heat and Triton X-100 affected the detection of VEGF similarly. The detection of IL-1α (−54 to −92%), IL-16 (−34 to −97%), IL-15 (−62 to −88%), IL-12p40 (−77 to −99%), and VEGF (−24 to −33%) was noticeably impacted in at least two sample types that were inactivated by heat (Fig. 3). A list of analytes that were significantly affected by heat or Triton X-100 inactivation (Table S1a) and a list of analytes that were not affected by either of the two inactivation methods (Table S1b) can be found in the supplementary material.

### Heat inaction had a more prominent impact on the detection of cytokine/chemokine than the Triton X-100 treatment

When comparing aliquots of the same sample that were either treated with heat, or 0.5% Triton X-100 or left untreated on ice, the magnitude of effects of heat-inactivation reached as high as 92% (IL-12p40, *p = 0.008) reduction in the detection. In contrast, the effects of 0.5% Triton X-100 on the detection of cytokine/chemokine did not exceed 37% (IL-15, p = 0.06). Moreover, while heat-inactivation affected the detection of analyzes in all 3 clinical samples, Triton X-100 only significantly affected the 5 analytes in the UTM of NP swab. The effects were mild, shown in greyish orange color (Fig. 3). Heat inactivation impaired the detection of 8 analytes to a larger extend, shown in brighter blue and orange. IL-12p40, IL-15, IL-16, IL-1α, and VEGF were significantly affected by heat-inactivation in 2 of the 3 sample types. Detection of IL-15 was decreased by 62% (*p = 0.02) and 88% (*p = 0.008) in ETTA and plasma, respectively. However, heat had no significant impact on the detection of IL-15 in the UTM of nasopharyngeal swab, with a 6% decrease at a 95% confidence level (Fig. 3). Moreover, the detection of Eotaxin-3, IFN-γ, and chemokine, such as IP-10, MCP-4, MIP-1β, MIP-3α, TARdC and TNF-α in the plasma samples seemed to be greatly reduced by heat-inactivation (blue dots) but the effects were not statistically significant (p-values ranged 0.12 to 0.06). It is, perhaps, due to the small sample size analyzed, and low detection levels, which resulted in a larger CV. Samples with CV greater than 20% were not included in the analyses. Collectively, heat-inactivation method had greater impacts on the detection of more analytes in all sample types, while Triton X-100 affected mostly 5 analytes in the UTM of NP swabs. Analytes in ETTA/lung fluids were not impacted by Triton X-100 treatment.

### Cytokines that were most impacted by heat inactivation have more hydrophobic residues, higher on the instability index

Features of each cytokine/chemokine examined in this study were obtained from the Expasy Protparam^34^, isoelectric calculator^31^, and Swiss model^36–44^ (Tables 1, S2 and S3). The protein structure of the 6 cytokines, IL-15, IL-16, VEGF, IL-12p40, and IL-1α (in grey font), were compared against the other 36 cytokine/chemokine that were less affected by heat or 0.5% Triton X-100 to determine whether any of the proteins features could be used to determine protein vulnerability to heat or Triton X-100. In Table 1, the different shades of gray were used to indicate the predicted instability of the protein, based on the feature examined. Each column depicts a feature of the analyte (row) examined. For example, IL-31, IL-23α, IL-27α, IP-10, IL-6, and GM-CSF had higher value in instability index, compared to others and therefore, had darker grey shades. IL-15, IL-16, and VEGF which were most affected by heat inactivation (Table 1) also had high values in the instability index and were shaded in darker grey color. Moreover, instability could be predicted by the presence of a low proportion of charged amino acids. Analytes, such as IL-27β, IL-23α, MIP-1α, MIP-1β, TNF-α, IFN-ƴ, IL-13, TNF-β, and IL-1α consist of lower proportions of charged amino acids in their sequences and had darker shades. Of these, IL-1α was the only one that was affected by heat (Table 1). Hydropathicity is also being used to predict protein stability, based on the amino acid composition. With the lower values of the grand average of hydropathicity (GRAVY), IL-17A, IFNƴ, IL-10, Eotaxin-1, Eotaxin-3, and MCP-1 were deemed less stable and shaded in darker grey.

**Table 1:**
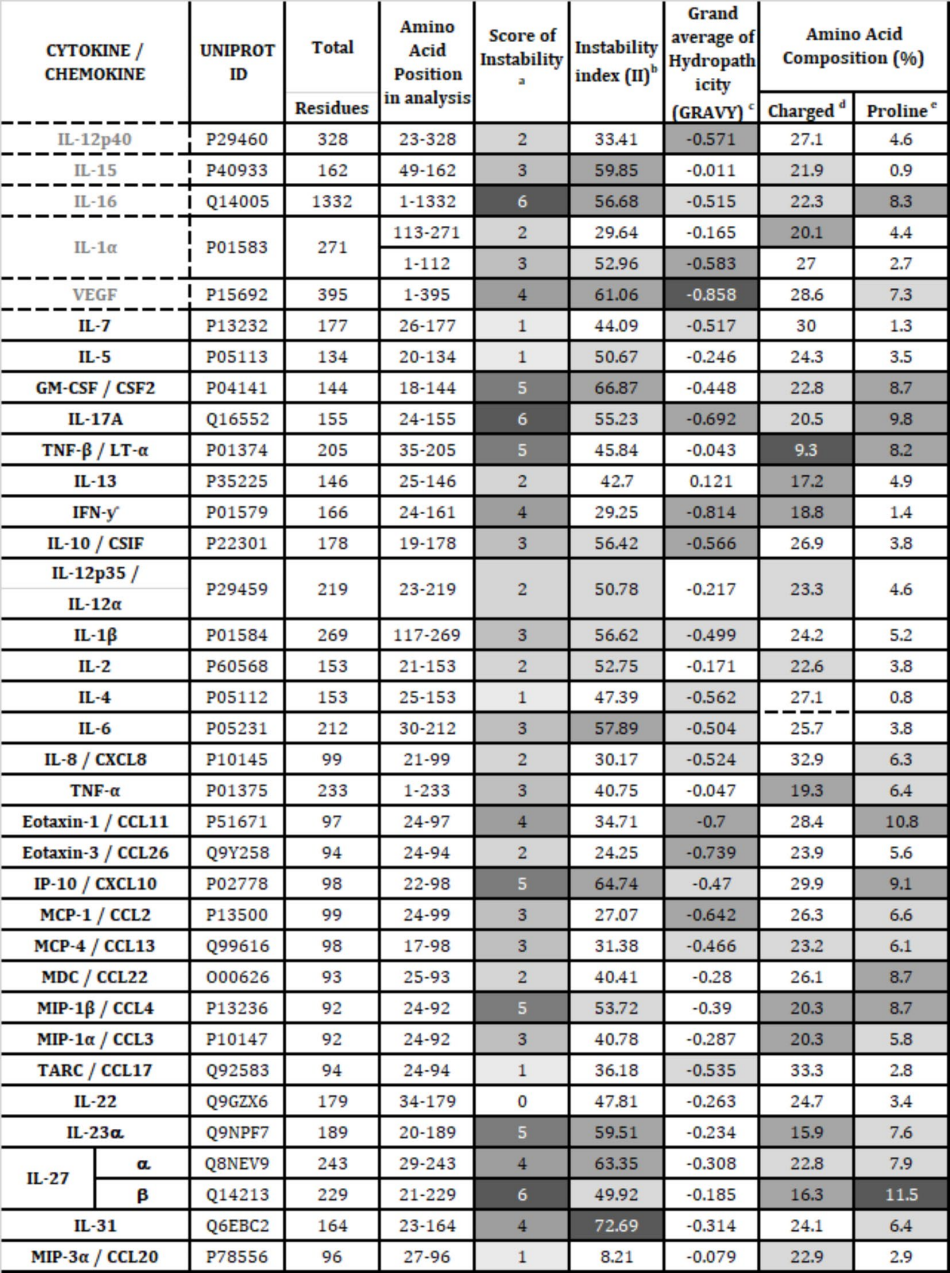
Instability factors of cytokine based on amino acid sequence.

Note: **Score of Instability**^a^: the score represents the sum of the grey shades in the columns to the right (protein features contributing to instability). , , , and □ were assigned of the scores, 3, 2, 1, and 0, respectively. The intensity of shading was based the values of the severity of instability features within the same column relative to the other cytokines. A grey-scale  to □ denotes the spectrum between the maximum value, “6”, and the minimum value, “0”.

**Instability index (II)**^b^ is a correlation between stability of a protein and its summation of a protein’s weight normalized to its sequence length. If the number of a protein is less than 40, then it is likely stable in a test tube environment (Guruprasad et al. 1990)^33^. , , , and □ denote the maximum value, 72.69, and values between 72.69 and 56.67, and between 56.67 and 48.87, respectively.

**Grand average of Hydropathicity (GRAVY)**^c^: is a spectrum that spans from −0.9 to 1.4, where low values indicate hydrophilic and high values, hydrophobic. GRAVY value is defined by the sum of hydropathicity values of all amino acids divided by the protein length (Gasteiger et al. 2005)^34^ , , , and □ denote the minimum value of -0.858, and values between -0.85 and -0.565, and -0.565 to -0.457, respectively.

Charged^d^: Composition of charged amino acids considered Asp, Glu, Arg, and Lys amino acids. , ,  and □ denote the minimum value of 9.3, and values between 9.3 and 20.35, and 20.35 to 23.6, respectively.

Proline^e^: , , , □ denote the maximum value, 11.5, values 11.5 to 8.13, and 8.13 to 5.7, and values <5.7, respectively.

Together, these instability features, based on the cytokine/chemokine amino acid sequence had some predicative value for the effects of heat on these proteins. For example, IL-16, VEGF, and IL-15, which were most affected by heat (Table 1, and S2) had higher instability score. IL-16 has the highest instability score and is high on the instability index and composition of hydrophilic amino acids and proline, while having a low proportion of charged amino acid. However, some of the cytokine/chemokine, such as GM-CSF, IL-17A and TNF-β have high instability score but were only minimally affected by heat or 0.5% Triton X-100 (Table 1 and S1). Furthermore, IL-1α has a low instability score but its detection in all sample types were greatly impaired following heat-inactivation, yet has low proportions of charged amino acids and hydrophobic amino acids, and an estimated half-life of only 1.9 h^34^, and has many residues in unfavorable conformations (Table S2). Hence, these instability features alone could not accurately predict protein susceptibility to heat or Triton X-100 treatment.

## Discussion

Heat inactivation had a greater impact on the cytokines, perhaps via alteration of protein conformation, resulting in the loss of antibody-recognizing epitopes and hence, the loss of detection of cytokine/chemokine. Although protein sequences are used for predicting protein stability^33,45–47^, analysis of amino acid sequences and structure alone cannot be used for predicting the protein stability against heat or Triton X-100 treatment.

### Heat inactivation is more damaging than Triton X-100 on cytokine epitope structure but Triton X-100 can still affect proteinaceous molecules

Many molecular assays use antibodies for detecting protein and/or sugar. The epitopes that are recognized by antibodies are highly specific and are conformational dependent. Specifically in this study, the antibodies used in the meso scale discovery panels were developed against the native form of protein, as antigen (personal communication). Most of these antibodies are likely to be dependent on the conformation of the epitope. Unfortunately, the protein properties and structure examined in this study (Table 1 and S3) could not fully predict the stability of the analytes when subjected to heat or Triton X-100. However, it is clear that only 8 analytes out of 36 were affected and that the analytes that were affected by Triton X-100 were also affected by heat (Fig. 3). It suggests that the intrinsic properties of the analyte/protein are still responsible for the stability of the analyte against the heat or Triton X-100 exposure. Further detailed analysis of other protein properties is needed to determine what can be used to fully predict protein stability.

Heat-treatment has been reported to reduce protein-detection by denaturing protein structure and altering protein conformation^19,48^. Denatured proteins could lose the epitope recognized by the antibody^48,49^, resulting in reduced or no detection of the target protein. On the other hand, Triton X-100, a non-ionic detergent solubilizes lipids of the cellular and viral membranes and should have minimum effect on protein conformation^50^. Yet, early studies by *Duck-Chong* and *Clarke *et al. showed that Triton X-100 could alter protein conformation via interacting with the hydrophobic regions of the protein with its nonpolar tail and hence, disrupting the native intermolecular interactions that hold the protein in shape^51,52^. In this study, all analytes are soluble molecules and therefore hydrophilic. We observed that extremely low hydrophobicity (more hydrophilic, lower negative GRAVY indices) is associated with protein instability in plasma samples (VEGF, Table S2). A correlation analysis of the percent change in plasma cytokine/chemokine detection that was treated with Triton X-100 (0.5%) versus the GRAVY score resulted in a negative correlation (*Pearson* r = −0.34, p = 0.04, with the confidence interval, -0.6003 to -0.01130, Table S2). Such correlation was not observed in the ETTA or the NP samples, treated with Triton X-100 (0.5%). These data further support that these protein characteristics alone cannot be used to predict the instability of the protein structure. We hypothesized that the media, in which the protein is immersed, also affects the magnitude of the effects from the treatment.

### Protein stability may be influenced by its environment, such as the composition of lung fluid or serum

The extent and the significance of the effects of heat and Triton X-100 (0.5%) inactivation on analytes vary between sample types. Cytokines from the lung are more stable than those in plasma or UTM. IL-16 and VEGF in plasma and NP-UTM samples were all affected by heat inactivation (NP, 21-34%, *p < 0.05; plasma, 34–97%, *p < 0.05) but not in ETTA samples. Perhaps components in different sample types influence stability of the analytes against heat or Triton X-100. Lung fluid typically contains membrane-associated mucins (MAMs)^53,54^. MAMs are known to increase stability of tear film of the eye^55^. In agreement with our observations that analytes in the ETTA samples were less affected by inactivation compared to the same analytes in other sample types. Hence, the mucins present in sample may serve as a stabilizer for the proteins in the sample.

The analyte in the sample types with the high protein concentration were affected more by heat-treatment. It seemed counter-intuitive, we observed that the largest number of analyte with significant change in detection (IL-1α, IL-16, IL-15, and IFN-ƴ) were present in heat-treated plasma rather than NP-UTM or ETTA. Plasma has approximately 100-fold higher protein concentration (39–60 mg/mL) than NP-UTM or ETTA (0.002–10 mg/mL). However, there has been no evidence from literature to explain such an observation.

In a clinical setting, cytokines are emerging as biomarkers for patients with AIDS, varieties of cancer, infections (such as sepsis, meningitis, or tuberculosis), and autoimmune disorders^25,28,56–58^. Moreover, blood/serum/plasma samples are commonly collected for diagnostic purpose. Uses of antibody-dependent molecular assays for detection of soluble factors have been instrumental in helping explain clinical observations or symptoms, assess the patient inflammatory status, monitor disease progression, confirm or guide diagnosis and treatments, stratify patients, or determine prognosis^23,24,26,27^. Should hospitals laboratories report results based on heat-inactivated samples, particularly plasma samples with reduced cytokine detection, this could result in under or wrong diagnosis, poor treatment plans and more.

## Conclusion

Different inactivation methods pose different extent of effects on the detection of cytokine and chemokine. Heat inactivation was observed to have the most effects on the detection of proteins in plasma, lung fluids and the UTM used for NP swabs. Unfortunately, protein stability based on amino acid sequence and structure along cannot reliably predict the protein stability and the extent of effect by heat or Triton X-100. This article provides a list of analytes that are affected (Table S1a) or unaffected (Table S1b) by either heat or Triton X-100 in these studied sample types for reference when clinical diagnostic tests or research studies of infection involve the inactivation of plasma/lung fluids/NP swabs to be used in protein assays. Finally, the protein’s stability in different sample types that have not tested here will need to be assessed empirically.

## Supplementary Information

Supplementary Information.

## Data Availability

Data is provided within the manuscript or supplementary information files. The raw datasets used and/or analyzed for the current study are available from the corresponding author on reasonable request.

## Abbreviations

ETTA: Endotracheal tube aspirate; lung fluid
NP: Nasopharyngeal
ICU: Intensive care unit
ARDS: Acute respiratory distress syndrome
UTM: Universal transport medium
DF: Dilution factor
LOD: Limit of detection
II: Instability index
IPC: Isoelectric point calculator
GRAVY: Grand average of hydropathicity
MAMs: Membrane-associated mucins
