# Supplementary material for: Detectability of cytokine and chemokine using ELISA, following sample-inactivation using Triton X-100 or heat
Source: Sci Rep. 2024 Nov 5;14:26777. doi: 10.1038/s41598-024-74739-0 (PMC11538312; doi:10.1038/s41598-024-74739-0)
Supplement: Supplementary file 1 — Supplementary Information. [file 41598_2024_74739_MOESM1_ESM.pdf]

Supplementary Information

Table S1a. Summary of cytokine/chemokine that were affected by Triton X-100 or Heat.

Inactivation	p-Value	Lung Fluid (ETTA)	NP Swab in UTM	Plasma
60°C for 1 hour	$p \leq 0.005$	↓IL-12p40 / IL-12β	↓IL-16<br>↓IL-1α	↑TNF-β<br>↓IL-12p40 / IL-12β<br>↓IL-15<br>↓IL-16
	$0.005 < p \leq 0.05$	↓IL-15<br>↓IL-1α	↑VEGF / VPF<br>↑IL-7	↑VEGF / VPF<br>↓IL-1α
	$0.05 < p \leq 0.1$	↑IL-13	↓IL-15<br>↓TARC / CCL17	↓IL-1β<br>↓IL-5<br>↓IL-6<br>↓IL-7<br>↓IL-10<br>↓IFN-γ<br>↓TARC / CCL17<br>↓TNF-α / LT-α
0.5% Triton X-100	$p \leq 0.005$			↑VEGF / VPF
	$0.005 < p \leq 0.05$		↑IL-15<br>↑IL-16<br>↑IL-1α<br>↑VEGF / VPF<br>↑IL-7	
	$0.05 < p \leq 0.1$		↑IL-1β<br>↑IL-12p40 / IL-12β<br>↑IL-17A / CTLA8	↑IL-1β<br>↑IL-6<br>↑IFN-γ<br>↑TNF-α / LT-α

Footnote: Cytokine / chemokine included in the table displayed a percentage of change in detection from the untreated samples. Direction, alluded to by ↑ and ↓, refers to the whether change in detection from untreated analyte detection levels were increased or increased.

Table S1b. Summary of cytokine/chemokine that are unaffected by Triton X-100 or Heat

Inactivation	**Lung Fluid (ETTA)**	**NP Swab in UTM**	**Plasma**
60°C for 1 hour	IL-16, VEGF, IL-12p70, Eotaxin-1/CCL11, Eotaxin-3/CCL26, GM-CSF/CSF2, IFN-γ, IL-10/CSIF, IL-17A, IL-1β, IL-2, IL-22, IL-23, IL-27α, IL-27β, IL-31, IL-4, IL-5, IL-6, IL-7, IL-8/CXCL8, IP-10/CXCL10, MCP-1/CCL2, MCP-4/CCL13, MDC/CCL22, MIP-1α/CCL3, MIP-1β/CCL4, MIP-3α/CCL20, TARC/CCL17, TNF-α, TNF-β/LT-α	IL-12p40/12β, IL-12p70, Eotaxin-1/CCL11, Eotaxin-3/CCL26, GM-CSF/CSF2, IFN-γ, IL-10/CSIF, IL-13, IL-17A, IL-2, IL-22, IL-23, IL-27α, IL-27β, IL-31, IL-4, IL-5, IL-6, IL-8/CXCL8, IP-10/CXCL10, MCP-1/CCL2, MCP-4/CCL13, MDC/CCL22, MIP-1α/CCL3, MIP-1β/CCL4, MIP-3α/CCL20, TNF-α, TNF-β/LT-α	IL-12p70, Eotaxin-1/CCL11, Eotaxin-3/CCL26, GM-CSF/CSF2, IL-13, IL-17A, IL-1β, IL-2, IL-22, IL-23, IL-27α, IL-27β, IL-31, IL-4, IL-8/CXCL8, IP-10/CXCL10, MCP-1/CCL2, MCP-4/CCL13, MDC/CCL22, MIP-1α/CCL3, MIP-1β/CCL4, MIP-3α/CCL20
0.5% Triton X-100	IL-15, IL-16, IL-1α, VEGF/VPF, IL-12p40/12β, IL-12p70, Eotaxin-1/CCL11, Eotaxin-3/CCL26, GM-CSF/CSF2, IFN-γ, IL-10/CSIF, IL-13, IL-17A, IL-1β, IL-2, IL-22, IL-23, IL-27α, IL-27β, IL-31, IL-4, IL-5, IL-6, IL-7, IL-8/CXCL8, IP-10/CXCL10, MCP-1/CCL2, MCP-4/CCL13, MDC/CCL22, MIP-1α/CCL3, MIP-1β/CCL4, MIP-3α/CCL20, TARC/CCL17, TNF-α, TNF-β/LT-α	IL-12p70, Eotaxin-1/CCL11, Eotaxin-3/CCL26, GM-CSF/CSF2, IFN-γ, IL-10/CSIF, IL-13, IL-2, IL-22, IL-23, IL-27α, IL-27β, IL-31, IL-4, IL-5, IL-6, IL-8/CXCL8, IP-10/CXCL10, MCP-1/CCL2, MCP-4/CCL13, MDC/CCL22, MIP-1α/CCL3, MIP-1β/CCL4, MIP-3α/CCL20, TARC/CCL17, TNF-α, TNF-β/LT-α	IL-15, IL-16, IL-1α, IL-12p40/12β, IL-12p70, Eotaxin-1/CCL11, Eotaxin-3/CCL26, GM-CSF/CSF2, IL-10/CSIF, IL-13, IL-17A, IL-2, IL-22, IL-23, IL-27α, IL-27β, IL-31, IL-4, IL-5, IL-7, IL-8/CXCL8, IP-10/CXCL10, MCP-1/CCL2, MCP-4/CCL13, MDC/CCL22, MIP-1α/CCL3, MIP-1β/CCL4, MIP-3α/CCL20, TARC/CCL17, TNF-β/LT-α

Footnote: Cytokine / chemokine included in the table had a percentage of change in detection from untreated samples that was significantly insignificant with a P-value >0.1.

Supplementary Table S2. Percent change of the cytokine-detection differences from the untreated.

Analyte		Uniprot ID	Amino Acid position	Score of Instability [a]	Instability Index [b]	GRAVY [c]	Amino Acid Composition: charge	Amino Acid Composition: proline	Median change from untreated (%): ETTA Heat	ETTA Triton	NP Swab Heat	NP Swab Triton	Plasma Heat	Plasma Triton
IL-12p40 / 12β		P29460	23-328	2	33.41	-0.571	27.1	4.6	76.98**	0	0	5.95	98.77**	1.51
IL-15		P40933	49-162	3	59.85	-0.011	21.9	0.9	62.28*	11.42	6.02	36.69*	88.23**	2.11
IL-16		Q14005	1-1332	6	56.68	-0.515	22.3	8.3	69.61	28	33.94**	27.27*	96.90**	9.36
IL-1α		P01583	113-271	2	29.64	-0.165	20.1	4.4	64.90*	19.04	54.47**	16.67*	92.48*	13.68
			1-112	3	52.96	-0.583	27	2.7						
VEGF / VPF		P15692	1-395	4	61.06	-0.858	28.6	7.3	23.84	12.17	21.38*	11.13*	32.88*	25.54**
IL-12p70	p40 / β	P29460	23-328	2	33.41	-0.571	27.1	4.6	0	29.14	0.06	0	7.7	32
	p35 / α	P29459	196	2	50.78	-0.217	23.3	4.6						
Eotaxin-1 / CCL11		P51671	24-97	4	34.71	-0.7	28.4	10.8	35.58	31.31_{n}	9.93	5.52	32.57_{n}	23.03
Eotaxin-3 / CCL26		Q9Y258	24-94	2	24.25	-0.739	23.9	5.6	27.76	0.49	4.38	4.38	85.35	0.6
GM-CSF / CSF2		P04141	18-144	5	66.87	-0.448	22.8	8.7	14.59	0	0	0	0	0
IFN-γ		P01579	24-161	4	29.25	-0.814	18.8	1.4	57.23	54.16	34.14	0	94.81	4.24
IL-10 / CSIF		P22301	19-178	3	56.42	-0.566	26.9	3.8	3.38	44.34	64.94	50.74	96.17	40.75
IL-13		P35225	25-146	2	42.7	0.121	17.2	4.9	32.56	25.51	5.11	6.76	19.06	0
IL-17A / CTLA8		Q16552	24-155	6	55.23	-0.692	20.5	9.8	0	0	0	0	36.18	3.81
									22.21	22.21	59.62_{n}	0_{n}	46.02	24.52
IL-1β		P01584	117-269	3	56.62	-0.499	24.2	5.2	58	58.11	64.38	62.41	46.82	59.13
IL-2		P60568	21-153	2	52.75	-0.171	22.6	3.8	0.96	1.83	4.86	2.84	0	0
IL-22		Q9GZX6	34-179	0	47.81	-0.263	24.7	3.4	0	3.32	60.82_{n}	0	30.92	8.65
IL-23		Q9NPF7	20-189	5	59.51	-0.234	15.9	7.6	0	0	135.23_{n}	0	54.28	19.74
IL-27	a	Q8NEV9	29-243	4	63.35	-0.308	22.8	7.9	0	0	95.53_{n}	49.15	98.97	7.36
	b	Q14213	21-229	6	49.92	-0.185	16.3	11.5						
IL-31		Q6EBC2	23-164	4	72.69	-0.314	24.1	6.4	0	0	0_{n}	0_{n}	161.43	0
IL-4		P05112	25-153	1	47.39	-0.562	27.1	0.8	0.06	10.36	0	0	30.65	30.65
IL-5		P05113	20-134	1	50.67	-0.246	24.3	3.5	10.6	5.19	0	0	13.58	3.35
IL-6		P05231	30-212	3	57.89	-0.504	25.7	3.8	9.9	25.61	19.42	4.19	91.35	34.02
IL-7		P13232	26-177	1	44.09	-0.517	30	1.3	27.92	0	26.01*	28.15*	25.36	6.6
IL-8 / CXCL8		P10145	21-99	2	30.17	-0.524	32.9	6.3	6.85	0.78	12.86	13.56	46.43	7.56
	HA								2.95	18.28	6.29	55.29	0	0
IP-10 / CXCL10		P02778	22-98	5	64.74	-0.47	29.9	9.1	22.37	25.11	7.46	3.82	64.68	13.2
MCP-1 / CCL2		P13500	24-99	3	27.07	-0.642	26.3	6.6	6.03	77.49	2.85	3.14	34.96	12.22
MCP-4 / CCL13		Q99616	17-98	3	31.38	-0.466	23.2	6.1	44.45	3.13	15.02	18.29	90.18	5.49
MDC / CCL22		O00626	25-93	2	40.41	-0.28	26.1	8.7	21.46	3.13	13.09	48.56	59.08	10.28
MIP-1α / CCL3		P10147	24-92	3	40.78	-0.287	20.3	5.8	26.51	25.11	2.93	17.78	21.28	1.57
MIP-1β / CCL4		P13236	24-92	5	53.72	-0.39	20.3	8.7	48.34	49.29	23.3	18.46	84.67	24.06
MIP-3α / CCL20		P78556	27-96	1	8.21	-0.079	22.9	2.9	63.77	5.93	6.05_{n}	36.71_{n}	98.98	3.32
TARC / CCL17		Q92583	24-94	1	36.18	-0.535	33.3	2.8	60.13	20.13	12.76	10.52	77.37	6.32
TNF-α		P01375	1-233	3	40.75	-0.047	19.3	6.4	0.7	11.84	8.98	30.11	91.52	35.12
TNF-β / LT-α		P01374	35-205	5	45.84	-0.043	9.3	8.2	0	0	0	0	80.35**	0

*, **, ***, and **** refers to a significant p value ≤ 0.05, 0.005, 0.0005, 0.0001 respectively. Median values presented and non-parametric tests used because data sets that did not pass normality testing. Values denoted with an 'n' showed too few samples or sample pairs to run normality or significance tests. Absolute percent change presented in the table has no directionality and is reported as magnitude only. **Red** and **Blue** colour-scale is used to describe the size of absolute percentage changes, caused by **heat-treatment** or **exposure to 0.5% Triton X-100**. The scale begins with white depicting a median no change value for the analyte and the colours grow more intense until they reach the maximum value (affected the most) present in the column.

Score of Instability [a]: the score represents the sum of the yellow shades in the columns to the right (protein features contributing to instability). The intensity of shading was based the values of the severity of instability features within the same column relative to the other cytokines. **A yellow-scale** from dark yellow to white denotes the spectrum between the maximum score (6), and the minimum score of 0 of each parameter examined. The darker the color, the more instable the protein was predicted to be.

Instability index (II)[b] is a correlation between stability of a protein and its summation of a protein's weight normalized to its sequence length. If the number of a protein is less than 40, then it is likely stable in a test tube environment (Guruprasad et al. 1990) [1]. Colour scale from dark yellow to white denote the maximum value, 72.69, and values between 72.69 and 56.67, and between 56.67 and 48.87, respectively.

Grand average of Hydropathicity (GRAVY) [c]: is a spectrum that spans from −0.9 to 1.4, where low values indicate hydrophilic and high values, hydrophobic. GRAVY value is defined by the sum of hydropathicity values of all amino acids divided by the protein length (Gasteiger et al. 2005) [2]. Colour scale from dark yellow to white denote the minimum value of -0.858, and values between -0.85 and -0.565, and -0.565 to -0.457, respectively.

Charged [d]: Composition of charged amino acids considered Asp, Glu, Arg, and Lys amino acids. Colour scale from dark yellow to white denote the minimum value of 9.3, and values between 9.3 and 20.35, and 20.35 to 23.6, respectively.

Proline[e]: Colour scale from dark yellow to white denote the maximum value, 11.5, and values between 11.5 and 8.13, and 8.13 to 5.7, respectively.

Table S3. Protein Characteristics of cytokines.

CYTOKINE	UNIPROT ID	RAMACHANDRAN PLOT	ALPHAFOLD TERTIARY STRUCTURE PROTEIN MODEL
**GM-CSF / CSF2**	P04141	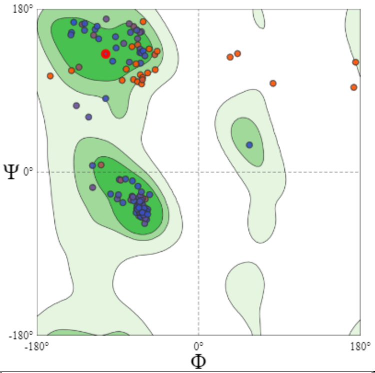	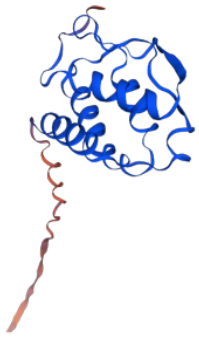
**IL-12p40 / IL-12β**	P294605JH	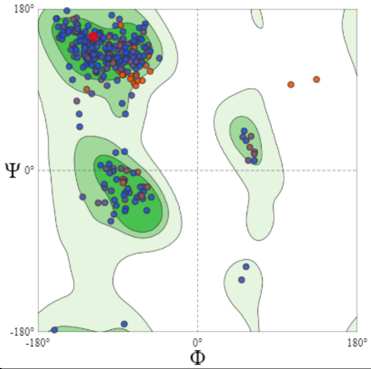	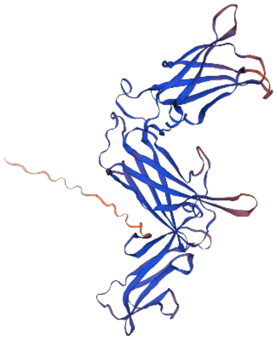
**IL-15**	P40933	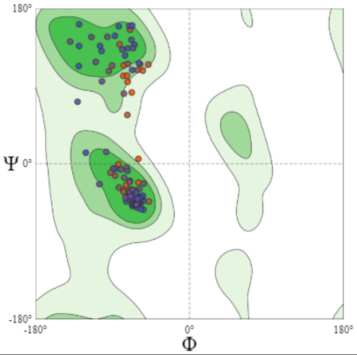	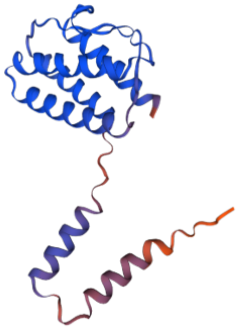

**IL-16**	Q14005	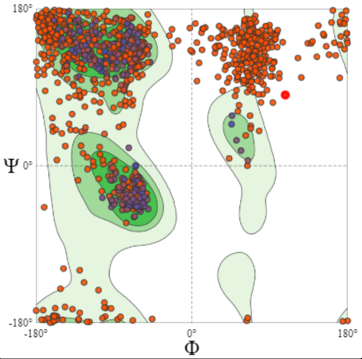	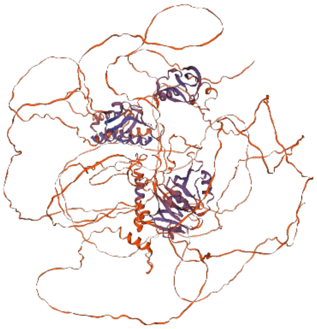
**IL-17A**	Q16552	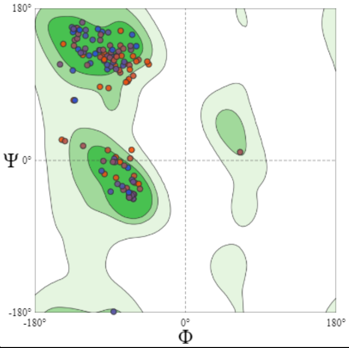	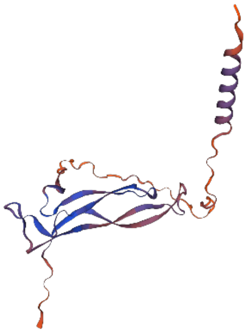
**IL-1α**	P01583	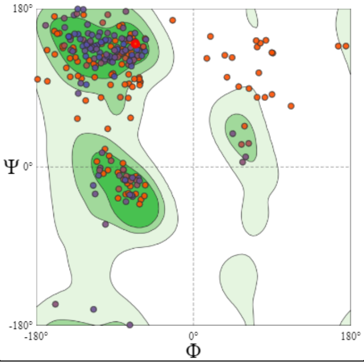	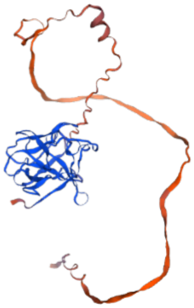
**IL-5**	P05113	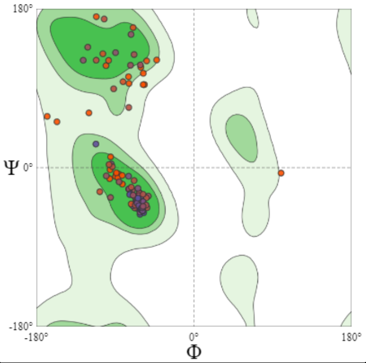	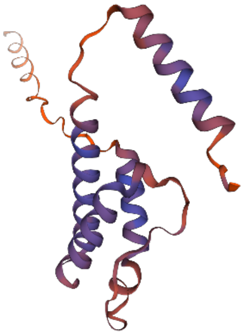

**IL-7**	P13232	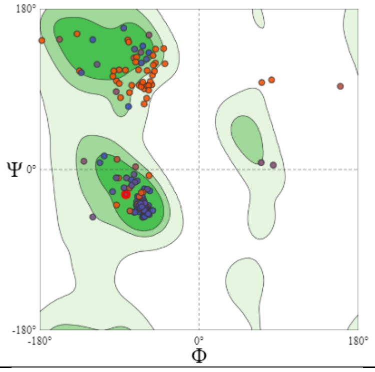	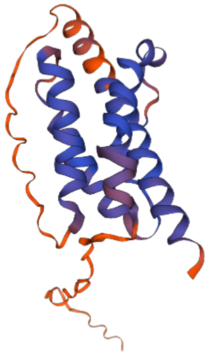
**TNF-β / LT-α**	P01374	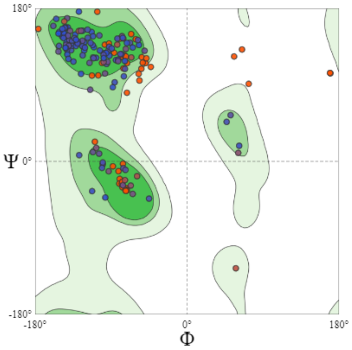	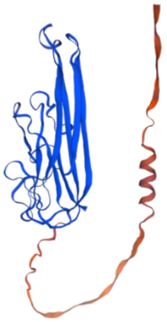
**IL-13**	P35225	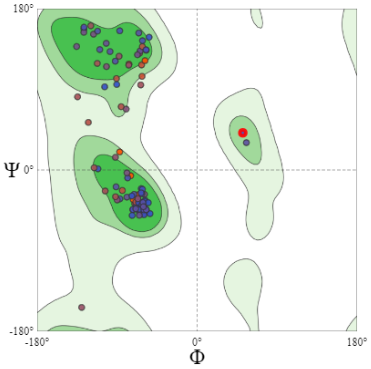	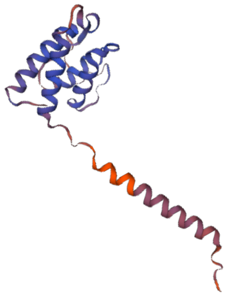
**VEGF**	P15692	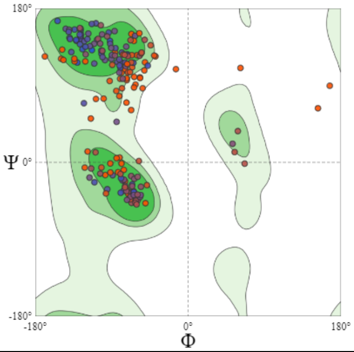	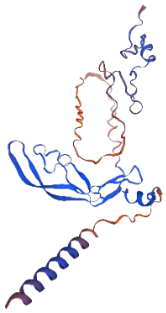

Each tertiary model has areas on the colour spectrum that reflects a per-residue confidence score (pLDDT) scores that range from low confidence (0) to high confidence (100). Dark blue, light blue, yellow, to orange indicates >90, 90-70, 70-50, and >50 on both the regions of the structure and dots on the Ramachandran plot. Unfavorable protein structure was not more common or consistent in repeatedly affected cytokines.

References:

1 Guruprasad, K. *et al.* Correlation between stability of a protein and its dipeptide composition: a novel approach for predicting in vivo stability of a protein from its primary sequence. *Protein Eng* **4**, 155-161, (1990).

2 Gasteiger, E. *et al.* in *The Proteomics Protocols Handbook* 571-607 (Humana Press, 2005).
